# Comparison of the Postoperative Effects of Local Antibiotic versus Systemic Antibiotic with the Use of Platelet-Rich Fibrin on Impacted Mandibular Third Molar Surgery: A Randomized Split-Mouth Study

**DOI:** 10.1155/2021/3040661

**Published:** 2021-12-02

**Authors:** Ceren Melahat Donmezer, Kani Bilginaylar

**Affiliations:** ^1^Department of Oral and Maxillofacial Surgery, Near East University Faculty of Dentistry, 99138, Nicosia TRNC Mersin 10, Turkey; ^2^Department of Oral and Maxillofacial Surgery, Final International University Faculty of Dentistry, 99320 Kyrenia, Cyprus, Turkey

## Abstract

The surgery of the impacted mandibular third molar is the most frequent procedure in dentistry. The prescription of systemic antibiotics after the third molar extraction is widespread among dentists, but this is still argumentative. This study is aimed at evaluating the postoperative effects of local antibiotic mixed with platelet-rich fibrin (PRF) and a postoperative systemic antibiotic prescribed for mandibular third molar surgery. The study included 75 patients divided into a control and 4 test groups (*n* = 15). In the control group, only PRF was placed into the extracted socket, and no antibiotic was prescribed. In the first and third groups, PRF was applied to the socket; penicillin and clindamycin were prescribed as oral medications, respectively. In the second and fourth groups, only PRF combined with penicillin and clindamycin was applied into the socket, respectively. The outcome variables were pain, swelling, analgesic intake, and trismus. These variables were also assessed based on the first, second, third, and seventh days following the operation. Unpaired Student's *t*-test and Mann–Whitney *U* test were used for analysis. There were significant differences in the total VAS pain scores between the control and group 3 (*p* < 0.05), groups 1 and 2 (*p* < 0.01), and group 4 (*p* < 0.001) in ascending order. For analgesic intake, there was no significant difference for group 1 (*p* > 0.05). However, there were statistical differences between the control group and groups 2 and 3 (*p* < 0.01) and group 4 (*p* < 0.001). Trismus and swelling did not differ among the groups (*p* > 0.05). This study showed that the effects of local and systemic antibiotics with the use of PRF reduced postoperative outcomes. Moreover, local antibiotics with PRF may be a viable method to avoid the possible side effects of systemic antibiotics.

## 1. Introduction

Postoperative complications of removal of impacted mandibular third molars, which is the most common and frequent dentoalveolar surgical procedure, may seriously compromise patients' recovery and quality of life [1, 2]. The most common complications associated with third molar surgery are pain, swelling, trismus, and secondary infections [2]. It has been previously reported that these complications negatively affect patients after surgical procedures [3]. As inflammatory complications remain a problem, numerous techniques have been attempted to decrease their incidence and accelerate postoperative healing [4]. These include platelet-rich plasma administration, cryotherapy, preoperative and postoperative antibiotics, osteotomy using high- or low-speed rotary instruments, wound drainage, the use of different flaps, postoperative ice packs, corticosteroids, analgesics, and lasers [5].

The practice of prescribing systemic antibiotics to prevent complications such as alveolitis and secondary infections is widespread among dentists. However, this remains a controversial topic in dentistry, as prophylactic antibiotic therapy is usually unnecessary in healthy patients. Improper use of antibiotics puts patients at risk for adverse reactions, such as allergic reactions, headaches, nausea, weakness, fever, diarrhoea, drug interactions, and gastrointestinal disturbances and also contributes to the development of antibiotic resistance [6–9]. In addition, there is little evidence in the literature regarding the benefits of preoperative and postoperative systemic antibiotics in dental surgery [10].

Because of its unique advantages, platelet-rich fibrin (PRF) application is an acceptable and popular procedure to accelerate soft and hard tissue healing. PRF is a second generation, autologous platelet concentrate that consists of leukocytes, platelets, cytokines, and circulating stem cells [11]. Instead of administering systemic antibiotics to patients, as suggested by Polak et al., an approach to combine antibiotics with PRF would be ideal [12]. To the best of our knowledge, no study has compared the postoperative effects of local antibiotic administration with PRF with those of systemic antibiotics in patients undergoing surgery for impacted mandibular third molars.

Therefore, this study is aimed at investigating and comparing the postoperative effects of local antibiotics combined with PRF clots with those of postoperative systemic antibiotics after surgical extraction of impacted mandibular third molars.

## 2. Materials and Methods

### 2.1. Ethical Considerations

The study protocol was approved by the Near East University Scientific Research Ethics Committee (project number NEU/2019/67-774). The trial was registered on “clinicaltrials.gov” (United States National Library of Medicine), with the identification number NCT04989127.

### 2.2. Study Setting and Grouping of Participants

This prospective, randomized, split-mouth study was conducted between March 2019 and February 2021 at the Near East University Faculty of Dentistry, Nicosia, Turkey. A total of 75 patients aged 18–40 years were included in the study. The criteria for patient selection were as follows: patients with no systemic diseases, history of opioid use for an extended period, current infections or acute pericoronitis, smoking or alcohol abuse, pregnancy, and known allergies to any antibiotic.

Patients with unilateral or bilateral vertically impacted mandibular third molars were selected according to the criteria mentioned above. In patients with bilateral impacted third molars, a minimum interval of 21 days between the two operations allowed parameters to return to baseline prior to the second surgery.

All included patients had the same surgical conditions regarding the inferior alveolar nerve, ramus, depth of impaction, and tooth position in the mandible. According to the classification system of Pell and Gregory, all mandibular third molar extractions were classified as moderately difficult operations (class I, level C) [13].

The selected third molars were divided into one control group and four test groups, including 15 surgical sites (*n* = 15) each. In the control group, only PRF was placed into the extraction socket, and no antibiotics were prescribed. In group 1, PRF was applied to the tooth socket and patients were prescribed an 875/125 mg amoxicillin/clavulanic acid tablet twice daily for five days. In group 2, PRF combined with 0.5 cc amoxicillin/clavulanic acid (1000/200 mg) was applied to the socket, and no systemic antibiotics were prescribed. In group 3, PRF was applied to the extraction socket; 300 mg clindamycin thrice daily for five days was prescribed as an oral medication. In group 4, PRF combined with 0.5 mL clindamycin 600 mg/4 mL was placed into the extraction socket; no systemic antibiotics were prescribed. All patients were instructed to take paracetamol 500 mg as an analgesic as required (every 4–6 h) and to use an antiseptic (7.5% povidone-iodine) mouthwash three times a day for 7 days. Pain, the number of painkillers taken, trismus, and swelling of the cheek were evaluated preoperatively and postoperatively on days 1, 2, 3, and 7. All examinations were performed at approximately the same time of day and by the same surgeon.

All patients were informed of the surgical procedure, postoperative time, and any possible complications. Patients were followed up on days 1, 2, 3, and 7; the surgical site was examined; local temperature was measured. None of the patients presented any signs of infection, such as redness (rubor), swelling (tumour), heat (calor), pain (dolor), and loss of function (functio laesa). Moreover, the volunteers stated that they experienced no signs of systemic disorders such as diarrhoea, nausea, headaches, and weakness.

### 2.3. Surgical Procedure

**Figure 1:**
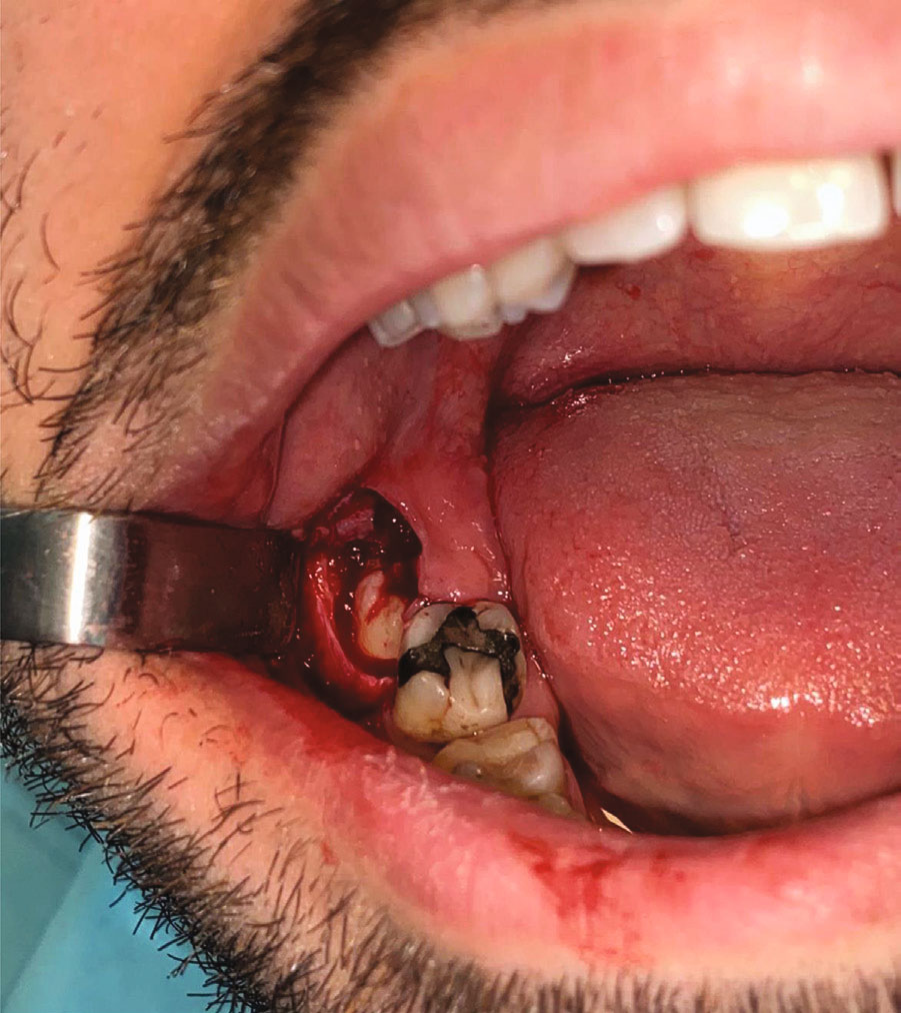
The same osteotomy technique was performed to remove the buccal bone around the crown of the impacted tooth in all groups. No tooth sectioning was performed.

All patients underwent radiological examination, including panoramic radiography, supervised by the same surgeon and assistant. To avoid muscle involvement, the flap was triangular in all groups (Archer's flap). Surgery was performed under local anaesthesia; inferior alveolar and lingual nerve blocks (regional anaesthesia) and buccal infiltration anaesthesia were performed using 40 mg/mL articaine HCl with 0.012 mg/mL epinephrine HCl (2 mL ultracaine D-S Forte Ampul; Sanofi Aventis). All operations were performed by raising a full-thickness mucoperiosteal flap. After mucoperiosteal flap reflection, the same osteotomy technique was performed to remove the buccal bone around the crown of the impacted tooth in all groups using a 1.6 mm round bur mounted on a W&H high-speed surgical handpiece at 40,000 rpm under abundant irrigation (Figure 1). No tooth sectioning was performed, and all parts of the tooth were loosened using an elevator prior to removal.

**Figure 2:**
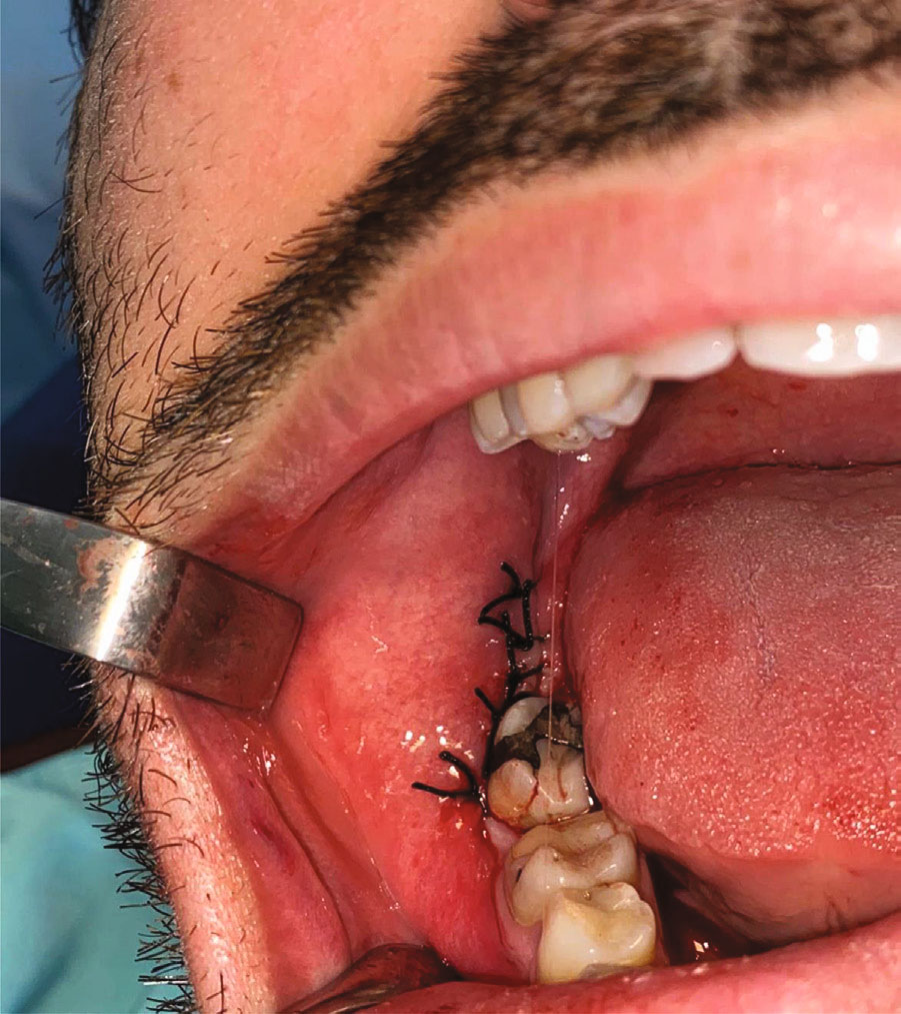
In all cases, PRF was placed gently without aspiration and the wound was immediately closed with 3-0 silk sutures.

In all cases, after removing the impacted tooth, the extraction socket was cleaned well using a sterile isotonic saline solution containing no antibacterial agent, before placing the PRF with or without antibiotics. PRF was placed gently without aspiration, and the wound was immediately closed with 3-0 silk sutures (Figure 2). A gauze pad was placed in the surgical area, and the patient was asked to bite down for 30 min. Sutures were removed after one week. Immediately after the operation, the patient was given an ice pack to apply every 10 min for six hours.

### 2.4. Preparation of PRF Gel and Antibiotic Administration

**Figure 3:**
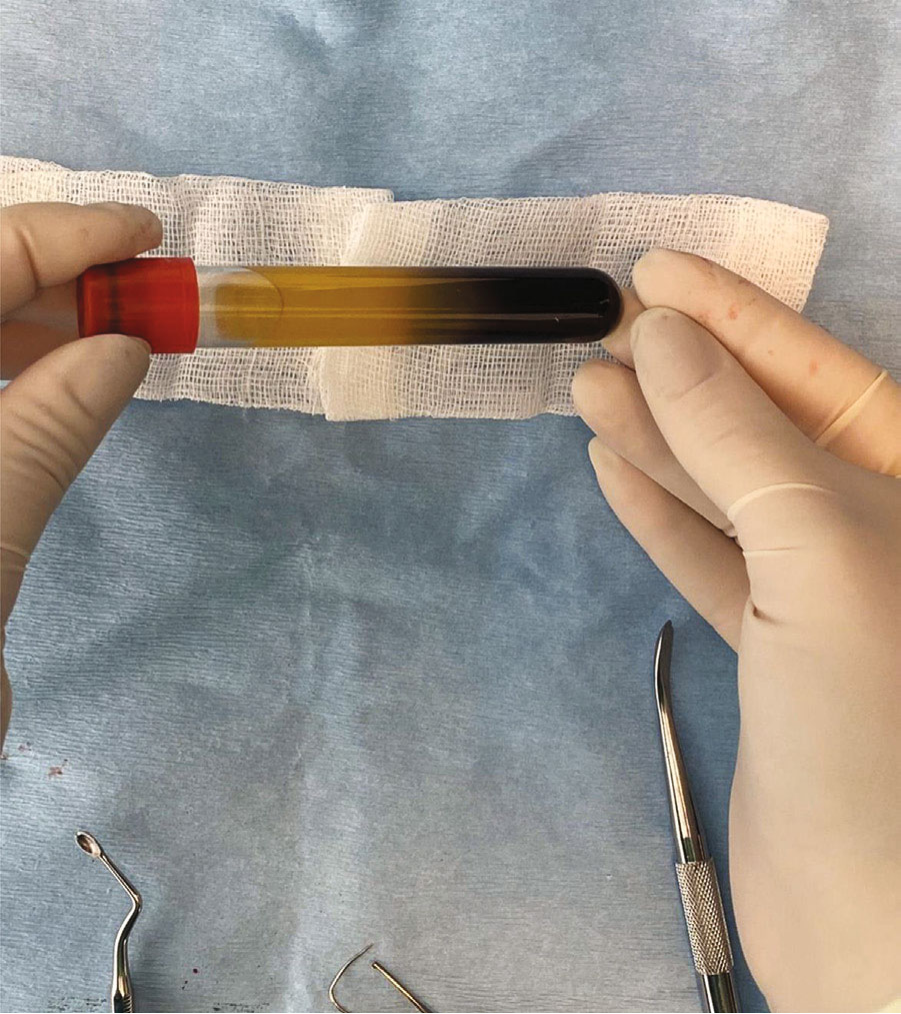
Each 10 mL tube produced one PRF clot, which was adequate to fill one extracted socket.

**Figure 4:**
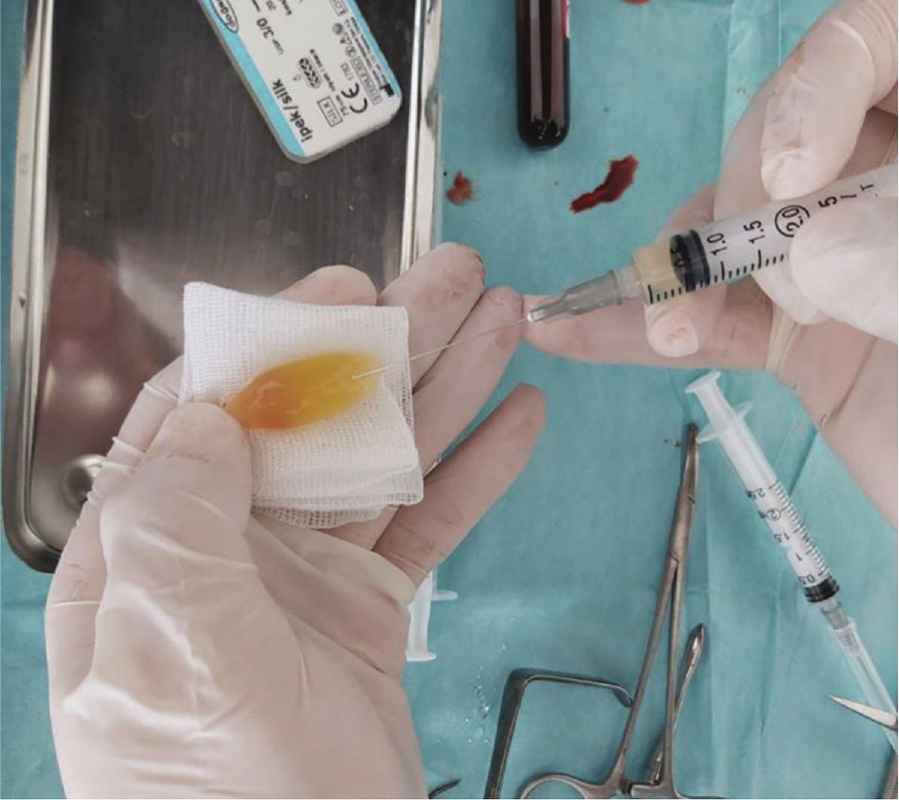
In groups 2 and 4, 0.5 mL antibiotics were injected into the PRF using a 2.5 mL dental syringe.

PRF was prepared according to the technique described by Dohan et al. Approximately 15 min before the surgery, a blood sample was taken in a 10 mL glass-coated plastic tube without anticoagulant. The sample was immediately centrifuged (Elektro-mag M415P) at 3,000 rpm for 10 min (approximately 400 g) [14]. The platelet-poor plasma that accumulated at the top of the tube was discarded. The PRF was dissected around 2 mm below its contact point with the red corpuscles situated beneath to include any remaining platelets that may have localised below the junction between the PRF and red corpuscles [15]. Each 10 mL tube produced one PRF clot, which was adequate to fill one extraction socket (Figure 3). However, in groups 2 and 4, 0.5 mL antibiotics were injected into the PRF using a 2.5 mL dental syringe (Figure 4).

### 2.5. Evaluation

We assessed postoperative pain using a visual analog scale (VAS) (0: no pain to 100: severe pain) [16] and recorded the number of analgesic tablets taken. We assessed trismus by measuring the distance between the mesial incisal corners of the lower and upper right incisors during maximum mouth opening, as described by Üstün et al., on postoperative days 1, 2, 3, and 7. Swelling was recorded using the modified method of Gabka and Matsumara described by Üstün et al. [17]. According to this measurement method, three preoperative measurements were taken between five reference points, the tragus to the soft tissue pogonion, the lateral edge of the eye to the angle of the mandible, and the tragus to the corner of the mouth, and were taken on postoperative days 1, 2, 3, and 7. The total of the three preoperative measurements was used as the baseline. The difference between each postoperative measurement and the baseline was taken as the value of facial swelling and trismus on that day [16]. Daily changes were recorded as percentages. The operating time was defined as the period between the first incision and completion of suturing. Patients were checked on each of the four postoperative days, and all measurements were assessed by the same person (not the operating surgeon) at roughly the same time of day.

### 2.6. Statistical Analysis

The GraphPad Prism software package was used for statistical analyses. Comparisons of VAS pain scores, trismus, and swelling between the groups were performed using unpaired Student's *t*-tests. The Mann–Whitney *U* test was used for intergroup comparisons of the number of analgesics taken. Statistical significance was set at *p* < 0.05. Data are presented as the mean ± standard deviation or median (Min–Max).

## 3. Results

All patients tolerated the medication well; there were no critical harm or unintended effects in any group. In addition, the patients reported no side effects of local and systemic antibiotic administration postoperatively.

Sex and operation side distributions did not differ between the groups (*p* > 0.05). Incorporating antibiotics into PRF required little time, and the duration of the surgery did not differ between the groups (*p* > 0.05).

### 3.1. Pain

On day 1, VAS pain scores in groups 1, 2, and 4 were significantly less than the control group (*p* < 0.001). However, VAS score in group 3 was not as less as the other test groups when compared to the control group (*p* < 0.05). On day 2, in groups 2–4, statistical differences in VAS pain score were similar to those on day 1 (*p* < 0.001, *p* < 0.05, and *p* < 0.001, respectively). In group 1, the VAS pain score difference increased compared to that in the control group on day 2 (*p* < 0.0001), changing from 18.33 ± 2.28 to 6.73 ± 1.73. On day 3, in groups 1–3, statistical differences were similar to those on day 2 (*p* < 0.0001, *p* < 0.001, and *p* < 0.05, respectively); however, in group 4, VAS pain scores changed from 9.32 ± 1.78 to 2.15 ± 0.69 (*p* < 0.0001). There were significant differences in the total VAS pain scores (sum of values on days 1, 2, 3, and 7) between the control and group 3 (*p* < 0.05), between the control and groups 1 and 2 (*p* < 0.01), and between the control and group 4 (*p* < 0.001), in ascending order (Table 1).

With regard to the amount of analgesic consumed by the patients, on day 1, there was no significant difference between groups 1 and 2 and the control group. However, the amount of analgesic consumed by patients in groups 3 and 4 was considerably less than that in the control group (*p* < 0.05). On day 2, there was no significant difference between the control group and groups 1 and 2, but in groups 3 and 4, the number of analgesic tablets consumed statistically decreased from 2.73 ± 0.25 to 1.40 ± 0.19 (*p* < 0.01) and from 2.47 ± 0.32 to 0.20 ± 0.24 (*p* < 0.05), respectively.

There was no difference in the total analgesic intake between the control group and group 3 on the third day; however, there was a significant difference between the control group and groups 2 (*p* < 0.001), 4 (*p* < 0.01), and 1 (*p* < 0.05), in descending order. In group 2, the number of analgesic tablets consumed changed from 2.01 ± 0.24 to 0.33 ± 0.13. There was no significant difference in the cumulative score (sum of values on days 1, 2, 3, and 7) between the control group and group 1. However, there were statistically significant differences between the control group and groups 2, 3 (both *p* < 0.01), and 4 (*p* < 0.001) (Table 2).

### 3.2. Trismus

There was no statistically significant difference in trismus between the control and test groups on day 1. On day 2, statistical differences were found between the control group and groups 1, 2 (both *p* < 0.0001), 3 (*p* < 0.01), and 4 (*p* < 0.05). On day 3, statistically significant differences were found between the control group and groups 1, 2, and 4 (*p* < 0.0001). In group 4, mouth opening changed from 41.07 ± 1.68 mm on day 2 to 38.13 ± 2.15 mm on day 3. However, the difference was lower in group 3 (*p* < 0.05) than in the control group. On postoperative days 1, 2, 3, and 7, there were no significant differences in trismus between the groups (Table 3).

### 3.3. Swelling

On day 1, there were similar significant differences between the control group and groups 2–4 (both *p* < 0.0001). In addition, there was a significant difference between the control group and group 1 (*p* < 0.01). On day 2, no differences were observed between the control group and groups 1 and 3. However, differences were found between the control group and groups 2 (*p* < 0.001) and 4 (*p* < 0.01). Swelling changed from 2.79 ± 0.30 mm to 2.36 ± 0.25 mm in group 2 and from 2.93 ± 0.34 mm to 2.91 ± 0.43 mm in group 4. On day 3, no differences were observed among any of the other groups. There were no significant differences in any of the parameters between the control and test groups on day 7 (*p* > 0.05) (Table 4).

## 4. Discussion

The problems most frequently encountered by patients after third molar surgery are swelling, pain, and trismus, which are used as parameters to evaluate the effects of systemic and local use of antibiotics after third molar surgery [18]. An essential point in the argument about antibiotics in third molar surgery is the administration of antibiotics, as possible side effects may occur after administration of systemic antibiotics. Some of the risks of antibiotic therapy include the toxicity of the substance itself, allergic reactions, secondary infections, and, most commonly, the development of resistant organisms. In general, clinicians show a tendency to overprescribe antibiotics and medications [19]. Prescription of systemic antibiotics is common after mandibular third molar surgery. However, it may be harmful and may affect the quality of life of patients. For this reason, new clinical trials are needed to identify different methods to support patients in the postoperative period [20].

This study is mainly aimed at comparing the effects of local antibiotics combined with PRF and postoperative systemic antibiotics after mandibular third molar surgery, for a minimum of 7 days. Till date, no clinical study has evaluated the effect of local antibiotic application combined with PRF in terms of pain, total number of painkillers required, swelling, and trismus after surgical extractions. PRF rapidly stimulates tissue healing by significantly increasing the recruitment and proliferation of various cells, including endothelial cells, gingival fibroblasts, chondrocytes, and osteoblasts, thereby heavily promoting tissue repair and angiogenesis at the site of injury [14, 15, 21].

In our study, PRF with or without antibiotics was used in the tooth socket. Beneficial results regarding the effects of PRF on wound healing have been reported previously; moreover, use of PRF can decrease the incidence of bacterial infections, such as osteomyelitis, following surgery, which are commonly reported following third molar extractions [22–26].

Previous studies have reported conflicting results regarding the use of systemic antibiotics. For example, Hellem and Nordenram compared the efficacy of antibiotics (lincomycin and penicillin V) and a local bandage (gauze sponge) saturated with Whitehead's varnish. They showed that the local bandage was significantly more effective than the antibiotics in preventing infection and, consequently, should be preferred, mainly because of the drawbacks of these drugs [27].

On the other hand, Mcgregor and Addy divided patients randomly into two groups: one group received penicillin, and the other group received a placebo. They reported that penicillin reduced trismus and swelling during the postoperative period [28]. In a randomized controlled clinical trial by Ren and Malmstrom, antibiotic therapy reduced alveolar osteitis and wound infection after third molar extraction [29]. Furthermore, according to a study by Monaco et al., antibiotic prophylaxis effectively prevented postoperative pain and wound infection after extraction of the mandibular third molars in young patients [30].

Our results regarding postoperative findings in the groups that received local and systemic antibiotics were in accordance with those of Mcgregor and Addy, Ren and Malmstrom, and Monaco et al.

Polak et al. conducted an in vivo study by incorporating antibiotics into PRF. They found that the addition of 0.5 mL of any of the tested solutions did not change the physical properties of the PRF. They showed that administered antibiotics may become trapped in the liquid phase of the PRF or within the PRF protein structure by testing PRF in its clot and pressed (membrane) forms, using three antibiotics: penicillin, clindamycin, and metronidazole [12]. According to their results, the antibacterial activity of clindamycin and penicillin against *S. aureus* was significantly higher than that of metronidazole during the four days of follow-up [12]. Therefore, in the present study, penicillin and clindamycin were used locally as antimicrobial agents in groups 2 and 4, respectively. The results showed that local administration of penicillin and clindamycin with PRF reduced postoperative complications.

## 5. Conclusions

In conclusion, this study showed that the outcomes of local and systemic antibiotic administration with the use of PRF after mandibular third molar surgery were similar, with both statistically reducing pain and analgesic intake. These procedures decreased trismus and swelling compared to those in the control group. Moreover, local administration of antibiotics with PRF may be a viable method to avoid the possible side effects of systemic antibiotics. However, to obtain accurate results, further randomized clinical trials are necessary.

## Figures and Tables

**Table 1:** The visual analog scale (VAS) pain scores of systemic and local applications in patients that underwent impacted mandibular third molar surgery.

	Control	PRF+amoxicillin (systemic)	PRF+amoxicillin (local)	PRF+CLİN (systemic)	PRF+CLİN (local)
VAS pain scores first day (mm)	33.87 ± 2.07	18.33 ± 2.28^∗∗∗^	17.66 ± 1.94^∗∗∗^	23.20 ± 2.83^∗^	18.87 ± 3.05^∗∗∗^
VAS pain scores second day (mm)	28.47 ± 3.56	6.73 ± 1.73^∗∗∗∗^	9.09 ± 1.11^∗∗∗^	13.31 ± 1.82^∗^	9.32 ± 1.78^∗∗∗^
VAS pain scores third day (mm)	16.69 ± 4.31	1.67 ± 0.78^∗∗∗∗^	2.83 ± 0.71^∗∗∗^	4.62 ± 0.61^∗^	2.15 ± 0.69^∗∗∗∗^
Total VAS pain scores (mm)	75.80 ± 9.24	34.57 ± 5.65^∗∗^	32.55 ± 4.36^∗∗^	39.10 ± 4.36^∗^	30.35 ± 4.85^∗∗∗^

Note: Data are presented as mean value ± standard deviation (median) in millimeter. VAS: visual analog scale; PRF: platelet-rich fibrin. ^∗^*p* < 0.05; ^∗^*p* < 0.01; ^∗∗∗^*p* < 0.001; ^∗∗∗∗^*p* < 0.0001. Comparisons according to the control group.

**Table 2:** Number of analgesics taken for systemic and local antibiotic use of patients who underwent impacted mandibular third molar surgery.

	Control	PRF+amoxicillin (systemic)	PRF+amoxicillin (local)	PRF+CLİN (systemic)	PRF+CLİN (local)
Number of analgesics first day	3.80 ± 0.28	4.6 ± 0.36	3.01 ± 0.19	2.73 ± 0.25^∗^	2.47 ± 0.32^∗^
Number of analgesics second day	3.73 ± 0.68	2.20 ± 0.26	2.01 ± 0.24	1.40 ± 0.19^∗∗^	1.20 ± 0.24^∗∗∗^
Number of analgesics third day	1.87 ± 0.36	0.67 ± 0.25^∗^	0.33 ± 0.13^∗∗∗^	0.80 ± 0.15	0.47 ± 0.17^∗∗^
Total number of analgesics	8.87 ± 1.03	7.40 ± 0.56	4.87 ± 0.41^∗∗^	5.13 ± 0.45^∗∗^	4.27 ± 0.53^∗∗∗^

Note: Data are presented as mean value ± standard deviation (median) in millimeter. PRF: platelet-rich fibrin. ^∗^*p* < 0.05; ^∗∗^*p* < 0.01; ^∗∗∗^*p* < 0.001; ^∗∗∗∗^*p* < 0.0001. Comparisons according to the control group.

**Table 3:** Trismus values of systemic and local antibiotic use of patients who underwent impacted mandibular third molar surgery.

	Control	PRF+amoxicillin (systemic)	PRF+amoxicillin (local)	PRF+CLİN (systemic)	PRF+CLİN (local)
Trismus (%) first day	40.05 ± 1.98	41.19 ± 1.99	38.38 ± 2.27	39.59 ± 1.96	42.86 ± 1.31
Trismus (%) second day	58.19 ± 2.57	34.61 ± 1.95^∗∗∗∗^	37.43 ± 1.82^∗∗∗∗^	39.94 ± 1.49^∗∗^	41.07 ± 1.68^∗^
Trismus (%) third day	50.09 ± 1.93	36.39 ± 1.78^∗∗∗∗^	37.84 ± 1.61^∗∗∗∗^	42.19 ± 1.19^∗^	38.13 ± 2.15^∗∗∗∗^
Trismus (%) seventh day	46.58 ± 1.39	41.72 ± 1.71	43.43 ± 1.90	41.89 ± 1.76	42.18 ± 1.53

Note: Data are presented as mean value ± standard deviation (median) in millimeter. PRF: platelet-rich fibrin. ^∗^*p* < 0.05; ^∗∗^*p* < 0.01; ^∗∗∗^*p* < 0.001; ^∗∗∗∗^*p* < 0.0001. Comparisons according to the control group.

**Table 4:** Swelling values of systemic and local antibiotic use of patients who underwent impacted lower third molar surgery.

	Control	PRF+amoxicillin (systemic)	PRF+amoxicillin (local)	PRF+CLİN (systemic)	PRF+CLİN (local)
Swelling (%) first day	7.35 ± 0.64	4.14 ± 0.41^∗∗^	2.79 ± 0.30^∗∗∗∗^	3.58 ± 0.69^∗∗∗∗^	2.93 ± 0.34^∗∗∗∗^
Swelling (%) second day	6.01 ± 0.60	3.69 ± 0.49	2.36 ± 0.25^∗∗∗^	3.39 ± 0.49	2.91 ± 0.43^∗∗^
Swelling (%) third day	2.66 ± 0.42	1.22 ± 0.21	2.71 ± 0.51	2.43 ± 0.45	2.19 ± 0.41

Note: Data are presented as mean value ± standard deviation (median) in millimeter. PRF: platelet-rich fibrin. ^∗^*p* < 0.05; ^∗∗^*p* < 0.01; ^∗∗∗^*p* < 0.001; ^∗∗∗∗^*p* < 0.0001. Comparisons according to the control group.

## Data Availability

The data are part of Ph.D. thesis of the main author, Near East University.
